# Virions at the Gates: Receptors and the Host–Virus Arms Race

**DOI:** 10.1371/journal.pbio.1001574

**Published:** 2013-05-28

**Authors:** John M. Coffin

**Affiliations:** Department of Molecular Biology and Microbiology, Tufts University, Boston, Massachusetts, United States of America

## Abstract

All viruses need to bind to specific receptor molecules on the surface of target cells to initiate infection. Virus–receptor binding is highly specific, and this specificity determines both the species and the cell type that can be infected by a given virus. In some well-studied cases, the virus-binding region on the receptor has been found to be unrelated to the receptor's normal cellular function. Resistance to virus infection can thus evolve by selection of mutations that alter amino acids in the binding region with minimal effect on normal function. This sort of positive selection can be used to infer the history of the host–virus “arms race” during their coevolution. In a new study, Demogines et al. use a combination of phylogenetic, structural, and virological analysis to infer the history and significance of positive selection on the transferrin receptor TfR1, a housekeeping protein required for iron uptake and the cell surface receptor for at least three different types of virus. The authors show that only two parts of the rodent TfR1 molecule have been subject to positive selection and that these correspond to the binding sites for two of these viruses—the mouse mammary tumor virus (a retrovirus) and Machupo virus (an arenavirus). They confirmed this result by introducing the inferred binding site mutations into the wild-type protein and testing for receptor function. Related arenaviruses are beginning to spread in human populations in South America as the cause of often fatal hemorrhagic fevers, and, although Demogines et al. could find no evidence of TfR1 mutations in this region that might have been selected as a consequence of human infection, the authors identified one such mutation in Asian populations that affects infection with these viruses.

## Host Cell Dependency Factors versus Viral Restriction Factors

Recent research on a number of fronts is making clear the remarkable extent to which interactions with infectious agents have shaped the evolution of their hosts. In particular, the survival, replication, and spread of viruses depend on interaction with many normal cell components, sometimes referred to as “host dependency factors.” As a rule, these factors are proteins that the cell requires for some normal function, but that have been co-opted by a virus for the same or another function. A large number of such factors have recently been identified by screening small interfering RNA (siRNA) libraries for effects on HIV replication [1], and a few candidate genes so identified have been confirmed by direct experiments [2]. Another class of interacting cellular components includes “restriction factors,” which usually play no obvious role in normal function, but seem to exist for the sole purpose of interfering with the replication of one or more viruses. Recently identified factors restricting retrovirus infection include a number of cellular proteins that interact directly with some virion component to block a specific event in the viral life cycle, including reverse transcription/nuclear import, integration of viral DNA, and release of virions from the infected cells [3]. Many such proteins are encoded by a group of genes whose expression is induced by interferons.

## Host–Virus Interactions Change through Positive Selection

A characteristic of restriction factor genes—unlike genes encoding housekeeping functions, which usually evolve under purifying selection—is that they often exhibit periods of positive selection in their evolutionary history [4],[5]. Purifying selection is a result of the fact that mutations that alter amino acids in a protein (nonsynonymous mutations) are almost always deleterious, while mutational change between synonymous codons has relatively little effect on fitness. The extent of such changes in the evolutionary history of a gene can be estimated by phylogenetic analysis of that gene among related species, and calculation of the ratio of nonsynonymous to synonymous mutations (dN/dS ratio) at individual codons. As a rule, genes involved in normal functions display dN/dS ratios of much less than 1, reflecting removal of deleterious mutations by selection. By contrast, genes encoding restriction factors, which interact with viral components, are often characterized by dN/dS ratios (in the portion encoding the virus-binding site) greater than 1, which is the hallmark of positive selection. The reason for this behavior is that viruses are also constantly evolving to evade restriction factors, by selection of mutations in the sites that bind them or by more elaborate mechanisms, such as proteins (like Vif or Vpu in HIV) that inactivate the factors. Escape of the virus can then be followed by mutations in the host to restore the restriction function, leading to selection anew for virus escape, and so on, creating an arms race that sometimes ends when the virus or host goes extinct. Even if the virus is no longer extant, the mark of positive selection in restriction factor genes, at least the portion encoding those amino acids that interacted with the virus, provides a clue pointing to infection of an ancestral species [6].

One might imagine that such positive selection could also play out in the opposite direction in dependency factors, but, with the exceptions discussed below, there are few examples. One reason for the absence of an obvious arms race involving such factors may be that the virus interacts with them in the same way that normal cell components do, so there is no room for change without disrupting normal function. Factors involved in intracellular transport of viral components, gene expression, nucleic acid replication, and other functions may exhibit low dN/dS ratios for this reason. In addition, factors that provide indirect support for viral replication, such as enzymes of nucleotide metabolism, could also be expected to fall into this category.

## When Host Dependency Proteins become Viral Receptors

An exception to the apparent lack of positive selection in dependency factors is the interaction with viruses and their cellular receptors, in which signs of an evolutionary arms race have been apparent from classical genetic studies for many years, and for which a paper published in this issue of *PLOS Biology* [7] provides strong support based on elegant phylogenetic and virological evidence. For all viruses, infection of a host cell requires interaction of a surface component of the virus particle (virion) with a specific cell component, usually, but not always, a cell surface glycoprotein. While virologists always call such components “receptors,” the term is somewhat misleading to people in other fields of biology, since it implies that the function of the protein is to play some sort of active role in “receiving” the virus. Importantly, however, a virus receptor need not be a receptor in the sense of a protein that interacts with some ligand for the purpose of signaling, cell–cell interaction, or something else. As far as the virus is concerned, the role of its receptor in infection is to provide a point of attachment to a target cell and a signal that it is in an appropriate place to initiate the events leading to fusion with the cell membrane and entry of the virion components into a cell. These events vary somewhat in their specifics from one virus group to another, but they always involve rearrangement of virion surface proteins to interact simultaneously with the virion surface and the cell membrane and draw them together until they merge. Virion–receptor binding can lead to this rearrangement directly at the cell surface, or indirectly, by mediating uptake of the virion into endosomes, which fuse with lysosomes, leading to a reduction in pH, which in turn triggers the fusion reaction. In both cases, the receptor plays a passive role, not an active one.

## Evidence of the Host Arms Race with Retroviruses

The virus–receptor interaction is exquisitely specific, and a single amino acid change in the receptor can completely abrogate this activity. Thus, the presence of a specific receptor is often a critical determinant in the species specificity of virus infection, and the cell specificity of receptor expression can be a decisive factor in the disease specificity of a virus. For example, HIV uses the CD4 protein as a receptor. CD4 is a key molecule in immune signaling and is expressed on, among others, helper T cells in all mammals. AIDS is a result of a slow loss of these cells due to the effects of virus infection. Despite the widespread distribution of CD4 in mammals, HIV-1 can use only the homolog found in primates for entry.

In well-studied cases, such as HIV-1, the binding site used by a virus is in a different portion of the receptor molecule than the site of ligand binding, and mutations in the virus-binding site are likely to be of little consequence to the normal host function. These properties can set the stage for the same sort of arms race as occurs with restriction factors. Indeed, the first host genetic elements found to affect retrovirus infection were receptor polymorphisms in chickens that prevent infection with avian leukosis viruses (ALVs) [8]. Corresponding polymorphisms are found in ALV isolates from chickens, which can be divided into subgroups (A–E) based on receptor usage and related properties [9]. One of the receptor genes, *Tvb*, comes in at least four flavors, encoding resistance or sensitivity to subgroups B, D, and E in various combinations [10]. Most chickens are sensitive to B viruses and resistant to E; related birds of other species are uniformly sensitive to E and resistant to B viruses. All infectious endogenous (i.e., inherited as a germline provirus) ALVs belong to subgroup E and are capable of infecting cells from many birds, but not most chickens. This paradox reflects the inferred arms race initiated by entry of a subgroup E virus into chickens, followed by selection of resistant *Tvb* alleles, and then by evolution of viruses capable of using either the resistant forms of Tvb or another protein as receptor. A similar sort of evolutionary back and forth is also apparent in the endogenous murine leukemia viruses [11] as well as some other mammalian retroviruses.

## The Transferrin Receptor as a “Common” Viral Entry Receptor

**Figure 1:**
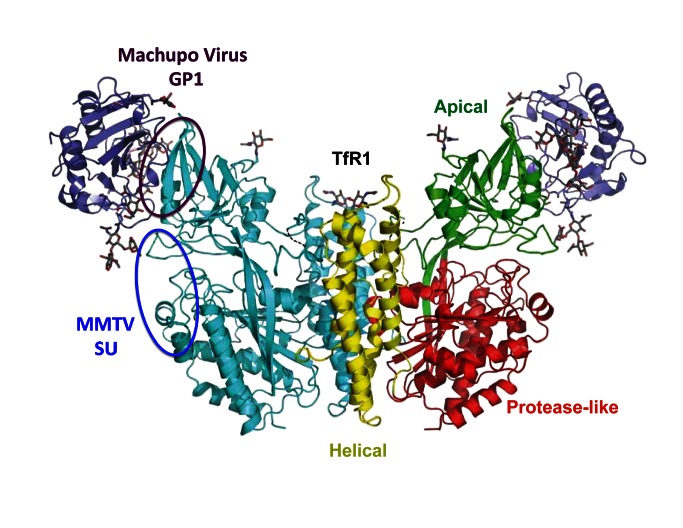
Structure of the transferrin receptor TfR1. The dimeric, butterfly-shaped protein TfR1 comprises three domains (colored yellow, red, and green in one protomer; the other protomer is shown in cyan). The receptor-binding domain of the Machupo virus GP1 protein (purple) is shown bound to the TfR1 apical domain. The purple and blue ovals illustrate the sites of Machupo GP1 and MMTV envelope surface (SU) protein binding, respectively, as well as the sites of positive selection. Reprinted with modification from [12], with permission.

Demogines et al. [7] applied sophisticated evolutionary, structural, and virological analysis to infer the arms race coevolution of another common mammalian cell surface protein and viruses that use it as receptor. The protein in question is TfR1, the receptor for iron-bound transferrin, which mediates iron uptake into cells. TfR1 is known to serve as receptor for viruses of three unrelated families: mouse mammary tumor virus (MMTV), a retrovirus; several rodent and human arenaviruses, such as Machupo; and parvoviruses, including canine parvovirus. In the latter two cases, evolution of the virus to use TfR1 in a different species has been a critical factor allowing recent spread of the viruses to humans and dogs, respectively. In the Machupo virus, the crystal structure of TfR1 bound to the virus GP1 entry protein reveals that the key binding site is a ridge in the apical portion of the butterfly-shaped receptor dimer [12] (Figure 1). Other lines of evidence have identified the binding site for MMTV as lying on an external ridge about halfway along the outside edge of the protein. To examine the details of the molecular coevolution of these two virus groups and their receptor, Demogines et al. used phylogenetic analyses to assess the dN/dS ratios of all extracellular TfR1 amino acids among a number of related rodents, including house mice (*Mus musculus*). Remarkably, only six residues exhibited ratios significantly greater than 1, and these mapped exactly to the MMTV and Machupo binding sites previously determined (Figure 1). Furthermore, when the same type of analysis was performed on the GP1 genes of Machupo virus and relatives, amino acids with dN/dS>1 were found to lie on the outward-facing side of the protein—at or near the sites of receptor binding. These results provide strong evidence for a back and forth coevolution involving the same housekeeping protein and between two different viruses and their rodent hosts. Interestingly, although arenaviruses are widespread, infectious MMTV is currently found only in *M. musculus*, and has not been described in any other rodent. Based on the evidence of positive selection for its binding sites, the authors speculate that it may once have had a much larger host range, but has become extinct in most lineages, perhaps by being unable to keep up with the resistant mutations in TfR1. Consistent with this idea, Demogines et al. identified closely related, but noninfectious, endogenous MMTV-like proviruses in a number of related rodent species, likely fossil remnants of the now extinct viruses.

## Viral Spread to New Hosts Can Be Determined by Mutations to Viral Entry Receptors

The paper concludes with an intriguing observation and an interesting evolutionary puzzle. Although most South American arenavirus infections occur in rodents, several such viruses are spreading into humans in the same region [13], so the authors examined human genome databases, seeking evidence for selection of mutations in TfR1 that might affect the emergence of this virus. Although no such mutation was found in South American DNA samples, Demogines et al. identified one polymorphic site (a substitution of valine for leucine at position 212) in Asian DNA sequences. Introduction of this mutation into the human TfR1 gene produces a modest (two-fold) decrease in its ability to serve as receptor for arenavirus in dog cells, which lack functional arenavirus receptors of their own. Intriguingly, the mutant TfR1 reduces the efficiency of infection of human cells by a small amount, even in the presence of a TfR1 allele that can serve as receptor for the virus. This observation raises the issue that mutations in virus receptors are expected to be recessive and thus have little or no effect on virus infection when they first arise, limiting the opportunity for positive selection to individuals homozygous for the mutation. Perhaps the expected two-fold reduction in active receptor concentration would provide a measure of protection sufficient to give a selective advantage to individuals carrying such a mutation. Alternatively, as the authors suggest, if the receptor protein is a multimer (dimer, in the case of TfR1), and all binding sites are essential for virus binding and entry, then such a mutation could be functionally dominant.

Given the evident importance of “new” viral diseases as they emerge into the human population, multifaceted approaches such as the one used in this paper promise to provide a valuable tool kit for understanding and preparing for their appearance.

## Abbreviations

ALV: avian leukosis virus
dN/dS ratio: ratio of nonsynonymous to synonymous mutations
MMTV: mouse mammary tumor virus
